# Intracranial phosphaturic mesenchymal tumor: A rare case report and systematic review

**DOI:** 10.1097/MD.0000000000041623

**Published:** 2025-02-21

**Authors:** Shuyue Song, Yuyang Zhao, Yiquan Wang, Yujing Zhao, Wenqiang Liu, Zhe Wang

**Affiliations:** a School of Clinical Medicine, Shandong Second Medical University, Weifang, China; b Department of Neurosurgery, Weifang People’s Hospital, Shandong Second Medical University, Weifang, China.

**Keywords:** hypophosphatemia, intracranial, osteomalacia, phosphaturic mesenchymal tumors, tumor-induced osteomalacia

## Abstract

**Rationale:**

Phosphaturic mesenchymal tumors (PMTs) are rare soft-tissue and bone tumors that can occur intracranially. Low incidence, nonspecific symptoms, and diverse histomorphology of PMTs contribute to a high rate of misdiagnosis.

**Patient concerns:**

This report presents a rare case of an intracranial PMT located in the posterior cranial fossa. In addition, a systematic review of previously reported intracranial PMT cases was conducted and summarized.

**Diagnoses:**

Incorporating clinical symptoms, laboratory findings, and imaging features, the definitive diagnosis of PMT was based on pathological examination.

**Interventions:**

The patient underwent consultations in endocrinology, orthopedics, and neurosurgery, and ultimately had a surgical procedure to remove the intracranial tumor.

**Outcomes:**

After tumor resection, the patient’s laboratory values returned to normal, his symptoms improved, and he could walk again.

**Lessons:**

Due to the rarity and high misdiagnosis rate of PMTs, no unified diagnosis and treatment standards have been established. Early identification, accurate diagnosis, and timely treatment are essential for optimal management. Surgical resection remains the preferred treatment for PMTs, with total tumor resection strongly recommended. In case of incomplete resection, tumor recurrence and persistent symptoms may necessitate adjunctive drug therapy and radiation therapy.

## 1. Introduction

Tumor-induced osteomalacia (TIO) is a rare paraneoplastic syndrome characterized by hyperphosphaturia, hypophosphatemia, and elevated alkaline phosphatase (ALP) levels.^[1]^ Most TIOs are caused by mesenchymal tumors,^[2,3]^ also referred to as phosphaturic mesenchymal tumors (PMTs).^[4]^ PMTs often present as osteomalacia, manifesting as pathological fractures, progressive muscle weakness, bone pain, and difficulty in walking. These symptoms are not directly related to the tumor itself.^[1,5]^

PMTs are mostly benign, rarely undergo malignantly transformation and metastasis, and are usually associated with fibroblast growth factor 23 (FGF23) overexpression.^[1,5,6]^ PMTs can occur in soft tissue or bone tissue. Soft tissues, are most commonly found in the extremities and acral sites, whereas bone tissues are usually located in the appendicular skeleton as well as head and neck regions. PMTs may occur in craniofacial areas, with the paranasal sinuses being the most common site; intracranial occurrences are rare.^[4]^ In this study, we report a rare case of intracranial PMT located in the right posterior cranial fossa. The patient exhibited hypophosphatemia and osteomalacia. We also reviewed the relevant literature.

## 2. Materials and methods

Informed consent was obtained from our study patient. Patient clinical information, including medical history, laboratory tests, pre- and postoperative imaging, pathological findings, and prognosis, was collected and analyzed.

Using the PubMed database, a comprehensive collection of data was performed to review all the articles on intracranial PMTs through January 2024. The search terms included “phosphaturic mesenchymal tumor” and “osteomalacia,” and all articles on intracranial PMTs were included. In addition, we screened the references of these articles, and sinuses or skull tumors with intracranial extension were also included. We collected information on the age, gender, tumor site, symptoms, disease duration, preoperative laboratory tests including the levels of serum phosphate, serum calcium, ALP, parathyroid hormone (PTH), 1,25-dihydroxyvitamin D (1,25-[OH] 2D), 25-hydroxyvitamin D (25 [OH] D), and FGF23, pathological findings, treatment, recurrence, and outcomes of all reported patients. The disease duration ranged from the date of symptom onset to the date of the first operation. The normal ranges and units for various laboratory tests varied in the reports. Depending on the reference ranges given in the reports, we replaced the values with normal, high or low labels. The data are displayed in Table 1.^[7–36]^

**Table 1:** Clinical features, diagnosis, and outcomes of intracranial phosphaturic mesenchymal tumor.

No.	Author/year	Age/sex (years)	Location	Size (cm)	Diagnosis	Symptoms	Osteomalacia	Serum phosphate	Serum calcium	ALP	1,25-(OH) 2D	25 (OH) D	Serum PTH	Serum FGF23	FGF23 expression in tumor	TFOTD (yr)	Treatment	Adjunctive therapy*	Outcome and recurrence (mo)
1	David et al (1996)^[7]^	60/F	Right frontal Anterior cranial fossa	–	PMT	Right hip pain, change in behavior and personality with increasing apathy, memory disturbance and bilateral anosmia	Yes	↓	Normal	↑	↓	↓	Normal	–	–	1.5	Surgery	No	Subtotal resection Recurrence (36)
2	Compta et al (1998)^[8]^	69/F	Right ethmoido-frontal extending to the anterior cranial fossa	–	PMT	Diffuse bone pain, progressive difficulty in walking and intracerebral hemorrhage	Yes	–	–	–	–	–	↑	–	–	19	Biopsy	No	Died (12 d)
3	Filho et al (2004)^[9]^	47/F	Left cavernous sinus	3.0*2.0*2.0	PMT	Muscle pain and weakness, visual disturbances and diplopia	Yes	↓	Normal	↑	–	–	–	–	–	7	Surgery	No	Subtotal resection Improved (48)
4	Kaylie et al (2006)^[10]^	46/F	Left temporal Posterior cranial fossa	–	PMT	Primary symptom: pulsatile tinnitus of left ear and vertigo Recurrent symptom: fullness of left ear, dizziness and bone pain	Yes	↓	–	↑	Normal		–	–	–	–	Surgery	No	Recurrence (24)
5	Yoshioka et al (2006)^[11]^	45/M	Left clivus region Posterior cranial fossa	–	PMT	Primary symptom: posterior neck and bone pain, left hypoglossal nerve palsy Recurrent symptom: painful motor impairment and multiple bone fractures	Yes	↓	Normal	↑	↓	–	Normal	Normal	–	2.5	Surgery	GKRS Octreotide therapy	Subtotal resection Recurrence (19)
6	Elston et al (2007)^[12]^	69/F	Frontal and parietal bones extending into the venous lakes and arachnoid granulation	–	PMT	Hypophosphatemia, bone pain and muscle weakness, wheelchair bound with a thoracic kyphosis and severe proximal myopathy	Yes	↓	–	↑	↓	Normal	↑	↑	Positive	5	Surgery	Octreotide therapy	Cured, NED (7)
7	Kobayashi et al (2011)^[13]^	53/F	Right temporal bone extending to the middle cranial fossa	–	PMT	Systemic bone pain and muscle weakness, vertebral and pelvis fractures	Yes	↓	Normal	–	–	–	–	↑	Positive	4	Surgery	Preoperative embolization	Cured, NED (13)
8	Uno et al (2011)^[14]^	53/F	Right temporal bone extending to the middle cranial fossa	2.0	PMT	Progressive body pain and unable to walk	Yes	↓	–	↑	–	–	–	↑	Positive	4	Surgery	Preoperative embolization	Cured, NED (2 wk)
9	Uno et al (2011)^[14]^	61/M	Left frontal Anterior cranial fossa	2.2	PMT	Primary symptom: body pain, difficulty in walking and intracerebral hemorrhage Progression: bed-ridden because of severe pain	Yes	↓	–	↑	–	–	–	↑	Positive	5	Surgery	No	Cured, NED (6)
10	Syed et al (2011)^[15]^	71/F	Right temporal bone extending to the middle cranial fossa	–	PMT	Primary symptom: hearing loss in the right ear Recurrent symptom: House–Brackmann grade IV, infranuclear facial nerve palsy on the right side	No	–	–	–	–	–	–	–	–	1	Surgery	No	Recurrence (12)
11	Chokyu et al (2012)^[16]^	57/M	Left temporal Middle cranial fossa	2.7*1.8*2.0	PMT	Bone pain and multiple bone fractures	Yes	↓	Normal	–	↓	–	–	↑	–	2	Surgery	No	Cured, NED (12)
12	Ling et al (2012)^[17]^	43/F	Right temporal bone extending to the sinus cavernous	1.7*1.3	PMT	Dffuse and progressive bone pain, muscle weakness, progressive dffculty in walking, esotropia in right eye and horizontal diplopia	Yes	↓	↓	↑	–	–	↑	–	–	4	Surgery	No	Subtotal resection Improved (8 d)
13	Bower et al (2012)^[18]^	67/F	Left frontal Anterior cranial fossa	7.4*4.3*5.1	PMT	Progressive abulia, apathy, depression, and urinary and fecal incontinence	No	–	–	–	–	–	–	–	Positive	1 mo	Surgery	No	Cured, NED (18)
14	Mathis et al (2013)^[19]^	28/F	Right frontal Anterior cranial fossa	1.8*1.8*1.0	PMT	Back stiffness upon exertion and proximal lower-extremity weak	Yes	↓	Normal	↑	↓	–	–	–	Positive	3	Surgery	No	Cured, NED (30)
15	Mathis et al (2013)^[19]^	32/M	Left frontal Anterior cranial fossa	–	PMT	Intracranial hemorrhage, hip and low-back pain	Yes	↓	–	–		–	–	–	Positive	13	Surgery	No	Recurrence (24)
16	Fathalla et al (2015)^[20]^	49/F	Right frontal Anterior cranial fossa	4.8*4.0*4.7	PMT	Multiple bone fractures	Yes	↓	Normal	–	↓	Normal	Normal	↑	–	3	Surgery	No	Cured, NED (6)
17	Ellis et al (2016)^[21]^	8/F	Right cerebellar hemisphere Posterior cranial fossa	–	PMT	Headache, unable to walk and ataxia	No	–	–	–	–	–	–	–	–	–	Surgery	No	Cured, NED (42)
18	Basu et al (2016)^[22]^	53/F	Left occipital Posterior cranial fossa	3.5*2.7	PMT	Bilateral groin pain and difficulty walking.	Yes	↓	–	–	–	–	–	↑	–	2	Surgery	PRRT	Subtotal resection First resection: recurrence (4) Second resection: recurrence (9)
19	Mulani et al (2017)^[23]^	48/F	Left temporal and occipital Posterior cranial fossa	–	PMT	Bilateral hips and thighs pain and progressive difficulty in walking, tinnitus and heaviness of the left ear	Yes	↓	Normal	↑	–	–	↑	↑	–	2	Surgery	Preoperative embolization	Cured, NED (1)
20	González et al (2017)^[24]^	42/M	Left nasal fossa extending to the anterior cranial fossa	8.0*4.0	PMT	Weakness and diffuse pain in the extremities, nasal obstruction	Yes	↓	Normal	↑	–	–	↑	↑	–	6	Surgery	No	Cured, NED (6)
21	Hana et al (2017)^[25]^	38/F	Right frontal, anterior cranial fossa extruding into bilateral ethmoid sinuses	–	PMT	Progressive bone, muscle pain and olfactory disturbance	Yes	↓	Normal	–	Normal	–	–	↑	Positive	7	Surgery (twice)	No	Cured, NED (25)
22	Ding et al (2018)^[26]^	68/F	Right frontal Anterior cranial fossa	–	PMT	Primary symptom: hypophosphatemia and Osteoporosis, no obvious bone pain Recurrent symptom: diffuse bone pain, frequent fractures	Yes	↓	–	–	–	–	–	–	–	–	Surgery	No	Recurrence (60)
23	Villepele et al (2018)^[27]^	41/F	Right nasal cavity and ethmoid sinus extending to the anterior cranial fossa	–	PMT	Multiple fractures, major disability and pain, nasal obstruction	Yes	–	Normal	–	–	–	Normal	–	–	–	Surgery	No	Cured, NED (9)
24	Mishra et al (2019)^[28]^	46/M	Right temporal Posterior cranial fossa	1.9*1.7*2.6	PMT	Progressive difficulty in walking and bilateral femoral fracture	Yes	↓	–	↑	↓	–	–	↑	–	5	Surgery	No	Cured
25	Mishra et al (2019)^[28]^	52/F	Left occipital Posterior cranial fossa	–	PMT	Bilateral hips and thighs pain and progressive difficulty in walking	Yes	↓	Normal	–	–	–	Normal	↑	–	3	Surgery	No	Cured, NED (3)
26	Walsh et al (2019)^[29]^	30s/F	Left cerebellopontine angle Posterior cranial fossa	1.5*2.0	PMT	Left tinnitus, distorted hearing, and occa sionalotalgia	No	↓	↓	–	–	–	–	–	Positive	–	Surgery	No	Subtotal resection Improved (1 d)
27	Gunawat et al (2019)^[30]^	53/F	Left jugular foramen Posterior cranial fossa	–	PMT	Bilateral hip, knee joint pain, and backache, proximal muscle weakness along with walking difficulty	Yes	–	–	–	–	–	–	↑	–	5	Surgery	Preoperative embolization	Cured, NED (6)
28	Gunawat et al (2019)^[30]^	45/M	Middle cranial fossa	–	PMT	Lower limbs and back pain	Yes	↓	–	–	–	–	–	↑	–	–	Surgery	No	Cured, NED (6)
29	Hadelsberg et al (2019)^[31]^	58/M	Left frontal Anterior cranial fossa	2.6*1.9	PMT	Osteoporosis, bone pain, muscle weakness, gait disturbances, and seizure	Yes	↓	Normal	–	–	–	–	–	–	6	Surgery	No	Cured, NED (2)
30	Colazo et al (2020)^[32]^	29/M	Right temporal Middle cranial fossa	1.3*1.1*1.0	PMT	Low-back and hip pain, gait changes, proximal muscle weakness, and multiple stress fractures	Yes	↓	Normal	↑	↓	–	↑	Normal	–	2	Surgery	No	Cured, NED (12)
31	Tang et al (2020)^[33]^	54/F	Right temporal bone extending to the middle cranial fossa	3.7	PMT	TIO-related symptoms, right tinnitus for and right ear hearing loss	Yes	↓	–	–	–	–	–	–	–	2	Surgery	Preoperative ICA stenting and embolization	Cured, NED (42)
32	Tang et al (2020)^[33]^	42/M	Right nasal cavity and ethmoid sinus extending to the anterior cranial fossa	3.5	PMT	TIO-related symptoms and hyposmia	Yes	↓	–	–	–	–	–	–	–	8	Surgery	Postoperative local radiotherapy	Subtotal resection First recurrence (24) Second recurrence (12) Died (24)
33	Riminucci et al (2022)^[34]^	39/F	Right nasal cavity and ethmoid sinus extending to the anterior cranial fossa	2.0	PMT	Muscular weakness, back pain, and fractures of vertebrae and hip, nasal obstruction and headache	Yes	↓	Normal	↑	↓	Normal	Normal	–	Positive	3	Surgery	No	Cured, NED (2)
34	Argersinge et al (2022)^[35]^	64/M	Right posterolateral frontal sinus extending to the anterior cranial fossa	3.0	PMT	Diffuse bone pain and weight loss	Yes	↓	Normal	↑	–	Normal	↑	↑	–	5	Surgery	No	Cured, NED (24)
35	Argersinge et al (2022)^[35]^	62/M	Right jugular foramen Posterior cranial fossa	2.4	PMT	Multiple fractures, diffuse bone pain and right-sided hearing loss	Yes	↓	Normal	↑	–	↓	↑	↑	Positive	–	Surgery	No	Cured, NED (30)
36	Kojima et al (2022)^[36]^	45/M	Right frontal Anterior cranial fossa	–	PMT	Right ankle and hip pain	Yes	↓	Normal	–	–	–	–	↑	Positive	–	Surgery	No	Cured, NED (120)
37	Present case (2024)	56/M	Right temporal and occipital Posterior cranial fossa	5.0*4.0*2.5	PMT	Headache, dizziness, multiple fractures and bone pain	Yes	↓	Normal	↑	–	↓	Normal	↑	–	8	Surgery	No	Subtotal resection Improved (3)

En dash indicates unavailable.

1,25-(OH) 2D = 1,25-dihydroxyvitamin D, 25 (OH) D = 25-hydroxyvitamin D, ALP = alkaline phosphatase, FEBI = focused external-beam irradiation, FGF23 = fibroblast growth factor 23, GKRS = gamma knife radiosurgery, ICA = internal carotid artery, NED = no evidence of disease, PMT = phosphaturic mesenchymal tumor, PRRT = peptide receptor radionuclide therapy, PTH = parathyroid hormone, SRT = stereotactic radiotherapy, TFOTD = time from onset to diagnosis.

* Oral medication therapy was excluded from adjunctive therapy because most patients took oral medications such as phosphates and vitamin D.

## 3. Results

### 3.1. Case report

**Figure 1.**
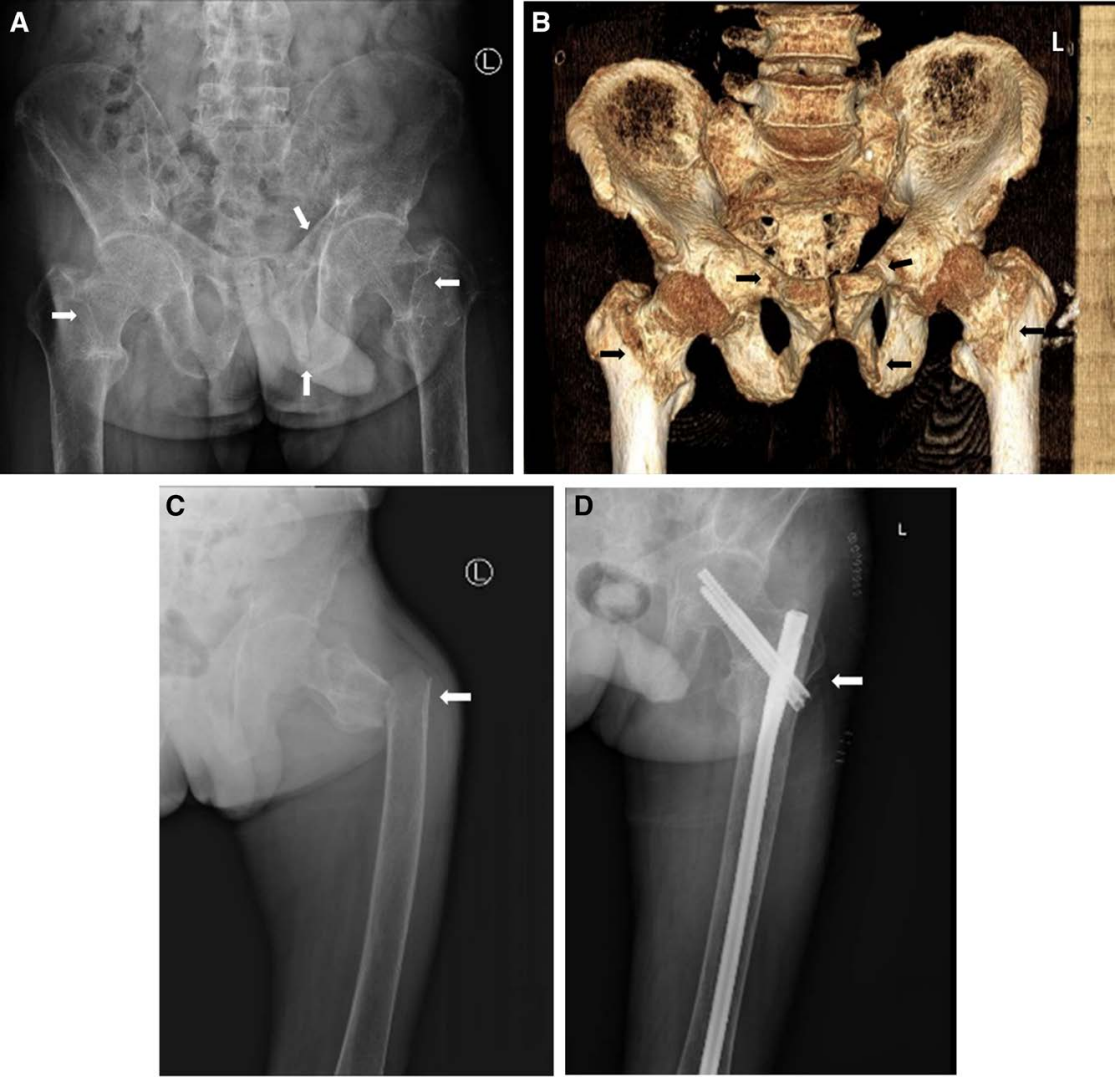
Pelvis radiograph (A) revealed pseudofractures of the bilateral femoral necks, irregular bone substance of the left superior and inferior ramus of pubis, and osteomalacia. computed tomography 3-dimensional imaging of the pelvis (B) showed bone discontinuity of the right superior ramus of pubis and left superior and inferior ramus of pubis, osteoporosis, nonhomogeneous density of the pelvis, and suspected fracture of the bilateral femoral necks. Fracture of the left proximal femur (C, left hip radiograph) occurring during hospitalization, with intramedullary fixation of fracture by open reduction (D, postoperative radiograph).

A 56-year-old male patient with a right temporo-occipital tumor was found 8 years ago due to experiencing headache and dizziness. Laboratory tests revealed elevated ALP (178 U/L, normal range: 45 to 125 U/L), normal serum phosphorus (0.85 mmol/L, normal range: 0.85 to 1.51 mmol/L), and normal serum calcium (2.43 mmol/L, normal range: 2.11 to 2.58 mmol/L). The doctor recommended that the patient undergo a biopsy, but the patient refused, and only 4 radiotherapies (200 cGy) were performed. After the completion of radiotherapies, the ALP level returned to normal (73 U/L), the serum phosphate level decreased (0.38 mmol/L), and the serum calcium level remained normal (2.24 mmol/L). Seven years ago, the patient developed generalized bone pain, which was prominent in the lower back and both lower limbs, and gradually worsened along with pain symptoms. Imaging tests revealed degenerative changes in the thoracic and lumbar spine and multiple old fractures of the bilateral ribs. Bone densitometry revealed severe osteoporosis. Laboratory tests revealed that ALP was 379 U/L and that serum calcium and serum phosphorus levels were normal. With long-term oral nonsteroidal anti-inflammatory drugs (NSAIDs) and traditional Chinese medicine, the pain was slightly reduced; however, the patient still experienced obvious pain. Muscular atrophy of the limbs subsequently occurred, and the patient gradually became unable to walk. Ten days ago, the patient’s bilateral hips pain increased, and the effect of oral medication was poor. Therefore, the patient was admitted to the endocrinology department of our hospital. Imaging tests revealed pseudofractures of the bilateral femoral necks. (Fig. 1) Laboratory tests revealed hypophosphatemia (0.32 mmol/L), elevated alkaline phosphatase (429 U/L, normal range: 45 to 125 U/L), 25 (OH) D deficiency (16.25 ng/mL, normal range: >30), and an elevated level of FGF23 (158.7 pg/mL, normal range: 23.3 to 95.4 pg/mL). Serum calcium and PTH levels were normal.

The patient was treated with calcium carbonate, calcitriol, vitamin D3, and NSAIDs in the endocrinology department. During this period, the patient suffered a fracture of the left proximal femur and was subsequently referred to the orthopedics department for surgical treatment (Fig. 1). The patient was highly suspected of having TIO due to the presence of hypophosphatemia and osteomalacia, and the intracranial tumor was thought to be the cause. The patient was finally referred to the neurosurgery department.

**Figure 2.**
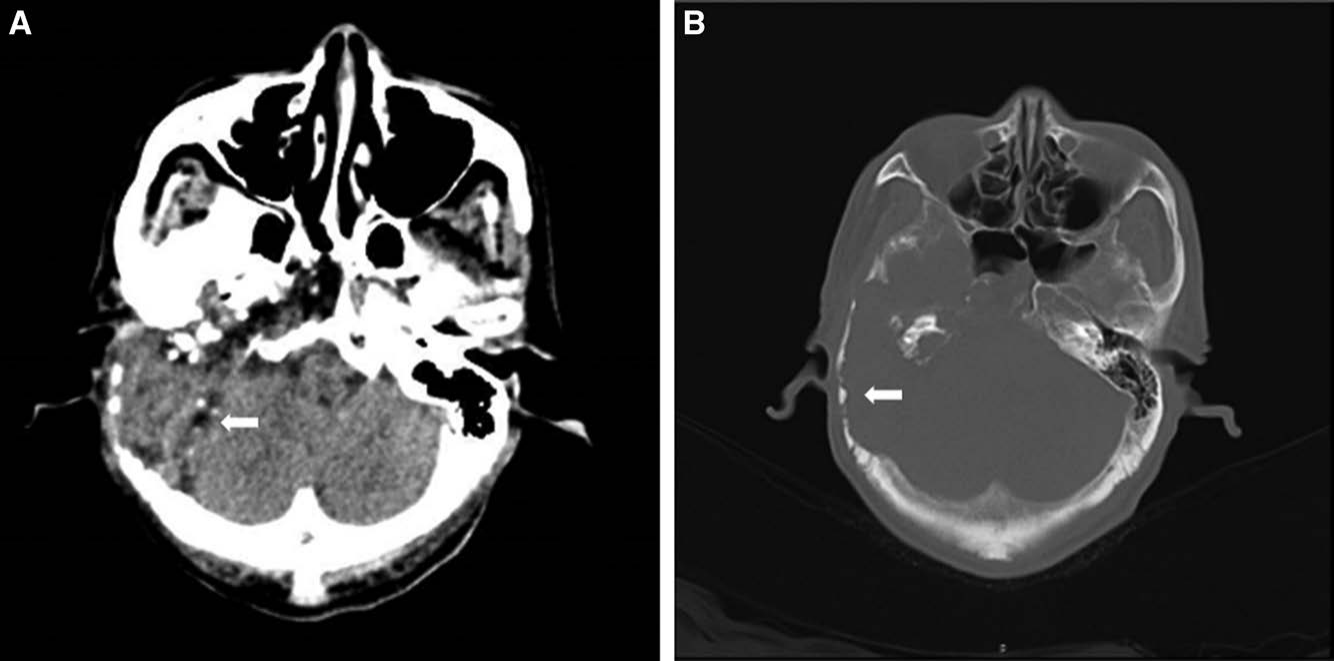
Preoperative computed tomography (A and B) revealed bone destruction of the right temporal bone, which was filled with soft tissue density, encroaching on the right inner and middle ear structures, encompassing the right internal carotid artery, partially entering the sphenoid sinus, and destroying the occipital bone posteriorly.

**Figure 3.**
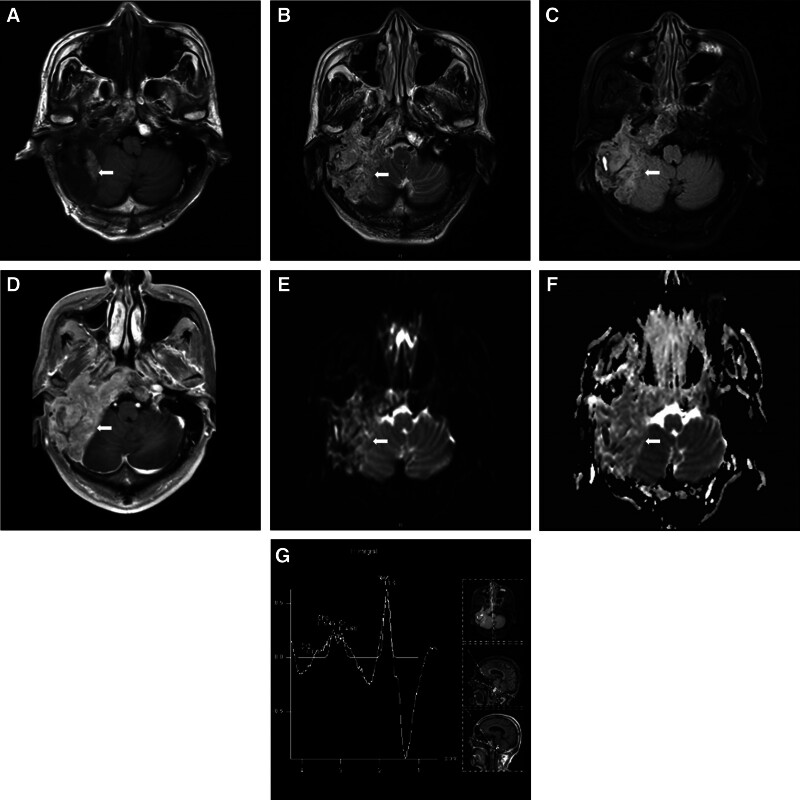
Preoperative magnetic resonance imaging revealed irregular heterogeneous T1 (A) and T2 (B) signals intensity in the right temporal bone and part of the sphenoid and occipital bone, heterogeneous signals intensity in the FLAIR sequence (C). The neighboring brain tissues were slightly compressed and displaced, and the fourth ventricle was compressed and deformed. After injection of GD-DTPA (D), large patchy abnormally enhanced areas were observed in the right sphenoid sinus area, clival, right temporal, petrous and pyramid areas; and right cervical area, with clear boundaries, and the right temporal area and right cerebellum were affected. Diffusion weighted imaging (E) and apparent diffusion coefficient (F) revealed partial restricted diffusion. The magnetic resonance spectroscopy (G) localization frame was located in the right temporal bone and part of the sphenoid and occipital lesions, with a TE = 135 ms, baseline instability, and waveform disorder. FLAIR = fluid attenuated inversion recovery, GD-DTPA = gadopentetic acid, TE = echo time.

**Figure 4.**
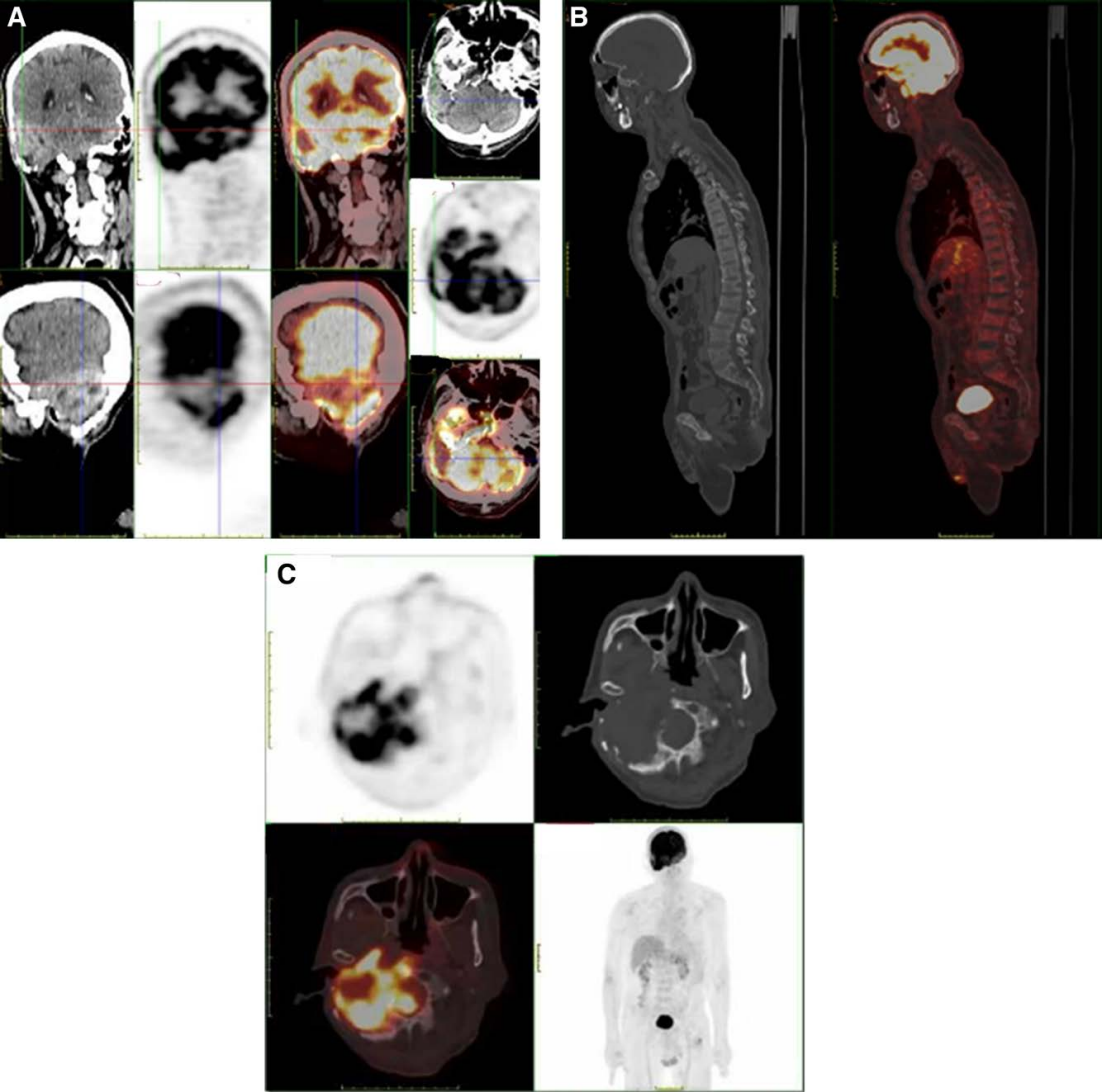
18F-FDG PET/CT (A and B) and 18F-OC PET/CT (C) revealed a mixed density mass in the right temporo-occipital region, with compression and displacement of the surrounding brain parenchyma, bone destruction of the right sphenoid, temporal bone and part of the occipital bone, and radioactive distribution was unevenly increased (18F-FDG PET/CT: SUVmax = 6.8, 18F-OC PET/CT: SUVmax = 10.9). Thoracic deformity was seen, with multiple ribs substance disorganization and osteotylus formation, and radioactivity distribution was mildly increased (18F-FDG PET/CT: SUVmax = 1.1, 18F-OC PET/CT: SUVmax = 2.0). Striped low-density shadows were seen in the bilateral proximal femur, and the radioactivity distribution of the left proximal femur was abnormally high (18F-FDG PET/CT: SUVmax = 7.1, 18F-OC PET/CT: SUVmax = 4.1). Decreased bone density in the skull, spine, pelvis, and extremities, multiple osteophyte formations in the lumbar spine, with no obvious abnormalities in radioactive distribution. 18F-FDG PET/CT results suggested that the right temporo-occipital mass after radiotherapy, inhomogeneous increase in metabolism, and the temporo-occipital tumor was considered the responsible lesion. 18F-OC PET/CT results suggested that the right temporo-occipital lesion had inhomogeneous high expression of octreotide, and the high probability of PMT. 18F-FDG = 2-deoxy-2-[fluorine-18] fluoro-d-glucose, 18F-OC = 18F-AlF-NOTA-octreotide, PET/CT = positron emission tomography/computed tomography, PMT = phosphaturic mesenchymal tumor.

Computed tomography (CT; Fig. 2) and magnetic resonance imaging (MRI; Fig. 3) revealed lesions occupying the right temporal bone and part of the sphenoid and occipital bones. To clarify the nature of the lesion, positron emission tomography with 2-deoxy-2-[fluorine-18] fluoro-d-glucose integrated with CT (18F-FDG PET/CT; Fig. 4) was also performed, revealing that the intracranial lesion was considered the responsible lesion. Notably, 18F-AlF-NOTA-octreotide (18F-OC) PET/CT results revealed a high probability of PMT.

The patient finally underwent surgery. During the operation, the tumor tissue was grayish-red, brittle and extremely rich in blood flow, and there were fragments of bone inside the tumor. After the tumor was removed, bleeding of the bone was obvious. The bone invaded by the tumor as well as parts of the temporal and occipital muscles were removed, and the intraoperative neurophysiological monitoring (IONM) did not indicate any obvious changes in the cranial nerves. Because part of the tumor protruded into the jugular foramen and the ventral aspect of the foramen magnum, it could not be completely removed. Postoperative radiation therapy was recommended for the patient.

**Figure 5.**
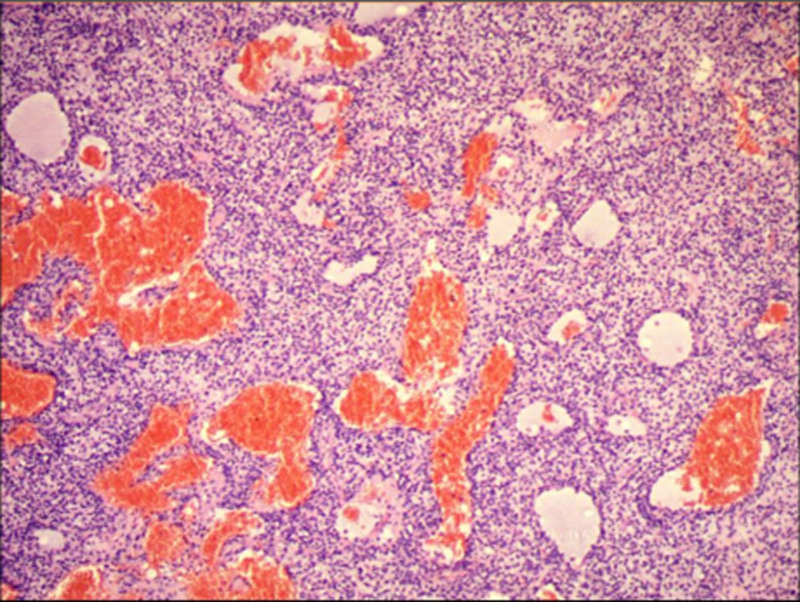
Pathological specimens: (The skull base lesion) A pile of grayish-white and grayish-red crushed tissue with a total volume of 5 cm × 4 cm × 2.5 cm. The cut surface was grayish-white, grayish-red, and had a soft texture. Pathologic diagnosis: Combined with immunohistochemical results consistent with PMT. PMT = phosphaturic mesenchymal tumor.

**Figure 6.**
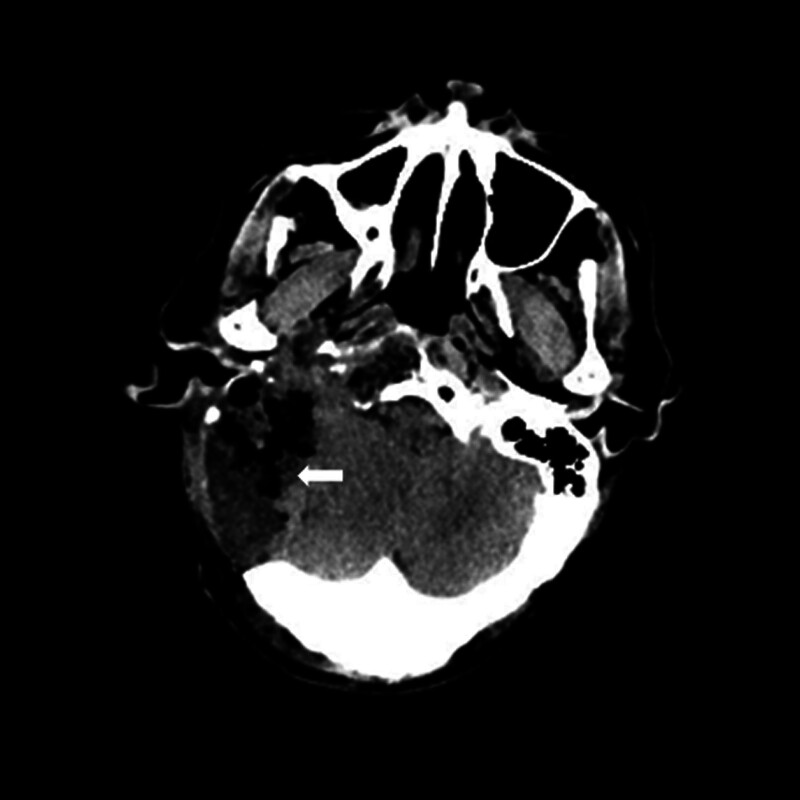
Postoperative computed tomography revealed partial loss of the right skull, with mixed density shadows and gas shadows in the operative area.

**Figure 7.**
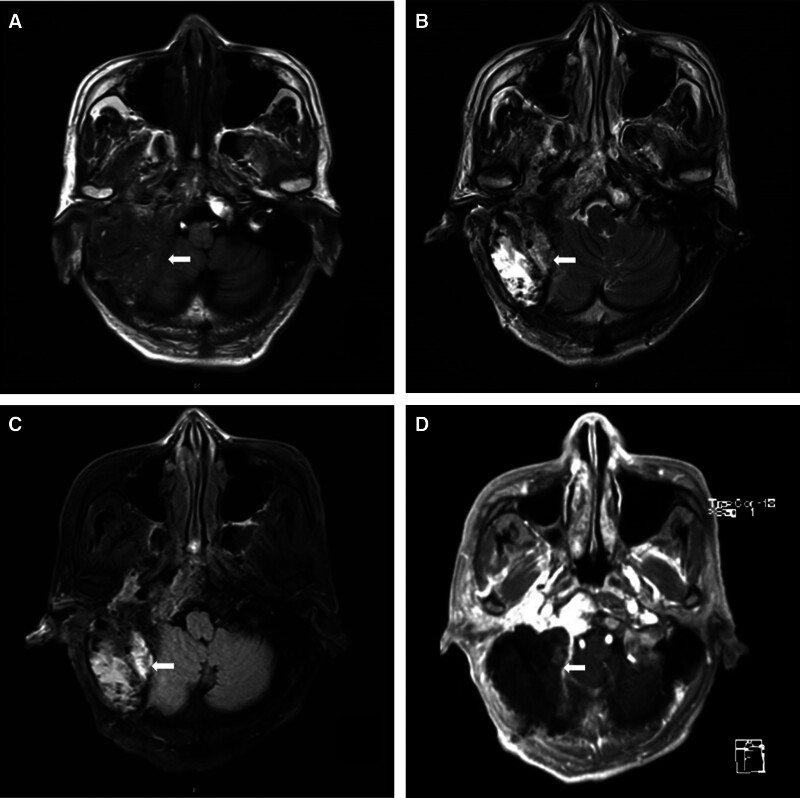
Postoperative magnetic resonance imaging revealed irregular heterogeneous T1 (A) and T2 (B) signal intensity in the operated area, heterogeneous signals intensity in the FLAIR sequence (C), and no obvious abnormalities was seen in the enhancement scan (D).

Postoperative pathological diagnosis (Fig. 5) suggested that the skull base lesion was consistent with a PMT. Immunohistochemical results revealed SSTR-2 (3+), Vimentin (+), CD56 (+), BCL-2 (+), CD99 (partially+), CK wide (−), PR (−), EMA (−), STAT6 (−), CD34 (vascular+), S-100 (−), NSE (−), and Ki-67 (index 5%). Postoperative imaging (Figs. 6 and 7) revealed that the patient had some residual tumor. The patient’s serum phosphate level returned to normal (0.86 mmol/L) after 2 weeks, and at the 3-month follow-up after discharge, the patient had recovered well, with significant improvement in physical strength. The patient had also resumed the ability to walk independently.

### 3.2. Systematic review

In our review, 37 patients with intracranial PMTs were described, including our current patient. The clinical data for all 37 patients are shown in Table 1. There were 14 (37.8%) males and 23 (62.2%) females and the average age at diagnosis was 48.50 years (range: 8 to 71 years). One case (2.7%) was diagnosed in a patient aged from 0 to 20 years, 6 cases (16.2%) were diagnosed in patients aged from 20 to 40 years, 22 cases (59.5%) were diagnosed in patients aged from 40 to 60 years and 8 cases (21.6%) were diagnosed in patients of more than 60 years. The most common site was the anterior cranial fossa in 16 cases (43.2%), followed by the posterior cranial fossa in 11 cases (29.7%), the middle cranial fossa in 7 cases (18.9%), the cavernous sinus in 2 cases (5.4%), and the frontoparietal bone in 1 case (2.7%).

Most patients had hypophosphatemia and osteomalacia. Except for 6 patients whose serum phosphate levels were not described, all patients had hypophosphatemia. Only 4 patients did not have osteomalacia (10.8%). Depending on the location of the tumor, a few local symptoms may present. PMTs in the anterior cranial fossa may present with anosmia (3 cases, 18.8%), nasal obstruction (3 cases, 18.8%), intracranial hemorrhage (3 cases, 18.8%), abulia and personality changes (2 cases, 12.5%), seizures (1 case, 6.3%), headache (1 case, 6.3%), and urinary and fecal incontinence (1 case, 6.3%). Middle cranial fossa PMTs may present with hearing loss (2 cases, 28.6%), tinnitus (1 case, 14.3%), and facial nerve palsy (1 case, 14.3%). PMTs in the posterior cranial fossa may present with tinnitus (3 cases, 27.3%), hearing loss (2 cases, 18.2%), vertigo (2 cases, 18.2%), headache (2 cases, 18.2%), ataxia (1 case, 9.1%), and hypoglossal nerve palsy (11 cases, 9.1%). Lesions in the cavernous sinus presented with visual disturbances and diplopia (2 cases, 100%).

Thirty-six patients were treated with surgery. Total tumor resection was performed in 28 cases; 24 cases had a good prognosis with no evidence of disease (85.7%), and 4 cases recurred (14.3%). Five of the patients who underwent total tumor resection were given preoperative embolization, and one was given octreotide therapy after the operation; all of these patients had a good prognosis and no evidence of disease. Case 21 underwent 2 surgeries before total tumor resection, with an interval of 2 years, and had a good prognosis with no evidence of disease. Subtotal resection of the tumor was performed in 8 patients, with improvement in 4 cases (50%) and recurrence in 4 cases (50%). Case 1 was not given adjunctive therapy except medication because the tumor did not regrow after recurrence. Case 5 received stereotactic gamma knife radiosurgery (GKRS) and octreotide therapy for recurrence, after which this patient’s symptoms improved. Case 18 relapsed 4 months after the first surgery and relapsed again 9 months after the second surgery. Because of recurrence and persistent symptoms after both surgeries, this patient was given peptide receptor radionuclide therapy (PRRT) combined with 177Lu-DOTATATE, and her symptoms improved after 3 months. Case 32 received postoperative local radiotherapy due to subtotal resection of the tumor. After 2 years, recurrence occurred, and the patient underwent surgery again. It recurred 1 year later and was resected again. The patient’s symptoms persisted, and he died of brain herniation 2 years later. Case 2, whose general condition was not suitable for tumor resection, underwent endoscopic biopsy only. This patient’s condition worsened after the biopsy, and he eventually died.

## 4. Discussion

Before 1987, the histologic features of PMTs were poorly understood, leading to varied diagnoses. In 1987, Weidner et al named these metabolic bone diseases, which cause osteomalacia, PMTs.^[37]^ In 2013, PMTs were included in the World Health Organization (WHO) for the first time and were classified as intermediate bone tumors with rare metastasizing behavior.^[38]^

The most common sites of PMTs are the extremities, particularly the lower extremities^[39]^; only 5% occur in the craniofacial region,^[14]^ with 61.9% of these in the sinuses.^[8]^ Intracranial PMTs most frequently occur in the anterior cranial fossa (63.2%),^[35]^ which is consistent with our findings. According to a study, PMTs are diagnosed between the age of 30 and 60 years, with a median age of 46 years. The male-to-female ratio was 0.7:1.^[3]^ In contrast, in head and neck PMTs, the female-to-male ratio was 1.6:1.^[8]^ In our review of intracranial PMTs, the age distribution was consistent with the general data, whereas the sex distribution showed a female predominance (1.7:1). We report the case of a male patient with a PMT located in the posterior cranial fossa, which is extremely rare in terms of both sex and site.

Unlike PMTs in general, intracranial PMTs may present with diverse local symptoms depending on the tumor site, in addition to typical osteomalacia-related symptoms such as fractures, bone pain, and muscle weakness. In our review, the most common symptoms of anterior cranial fossa PMTs confined to the cranium were abulia and personality changes (11.1%), as well as anosmia (16.7%) and nasal obstruction (16.7%) if the sinuses were involved. Middle cranial fossa PMTs most frequently cause hearing loss (28.6%), whereas posterior cranial fossa PMTs are often associated with tinnitus (27.3%). Lesions in the cavernous sinus frequently result in visual disturbances and diplopia (100%). Some patients may develop severe, life-threatening symptoms owing to tumor growth. We reviewed 3 patients who developed spontaneous intracranial hemorrhage, all in the anterior cranial fossa; one of these patients ultimately died. When evaluating patients with osteomalacia-related symptoms, it is crucial to consider potential local tumor-related symptoms. Neurological examinations should be promptly performed in patients showing central nervous system damage or infiltrative symptoms to enable early detection and treatment.

PMTs typically present as low serum phosphate, elevated ALP, and increased FGF23 levels. Among all patients with available data, 100% exhibited low serum phosphate and high ALP levels, whereas 91.0% had elevated FGF23 levels. PMTs are usually associated with the overexpression of FGF23, which inhibits the secretion of PTH and 1,25(OH)2D3. This disruption affects phosphate metabolism, leading to decrease in the intestinal reabsorption and urinary excretion of phosphate. This condition results in hypophosphatemia, which can lead to osteomalacia, manifesting as progressive muscle weakness, bone pain, and fractures.^[40,41]^ In addition to FGF23 levels, tubular reabsorption of phosphate and the ratio of tubular maximum reabsorption of phosphate (TmP/GFR) are crucial for the diagnosing PMT and distinguishing between reduced phosphate absorption and renal phosphate loss – key factors that are currently lacking in our case. Although there is no doubted in diagnosis, the clinical data remains incomplete. Moreover, we only reported the patient’s 25 (OH) D level and lacked the 1,25(OH)2D3 level, which is something we need to improve. Ensuring comprehensive data collection will enhance our ability to accurately diagnose and manage the disease.

Notably, PMTs often have nonspecific symptoms, are usually small, and occur in specific locations, making them challenging to detect and localize. Consequently, misdiagnosis and delayed diagnosis are common, often resulting in failure to provide accurate and timely diagnoses. In our review, the average delay before the diagnosis of PMTs was approximately 5 years. The definitive diagnosis of PMTs require a comprehensive evaluation, including detailed medical history, physical examination, laboratory tests, and imaging findings. When patients present with unexplained hypophosphatemia and osteomalacia, common causes such as vitamin D deficiency should first be excluded. A comprehensive physical examination should follow to identify potential tumors and confirm the diagnosis of PMTs. Imaging findings are particularly important.

Currently, the main imaging tools for the diagnosis of PMTs are CT, MRI, PET-CT, and octreotide imaging. In 2013, a study by Michelle Houang et al demonstrated that PMTs express various surface receptors, making octreotide imaging a useful diagnostic tool.^[42]^ Similarly, in a study by Gupta et al,^[39]^ 8 cases where octreotide scanning (DOTANOC-PET/CT) was performed, all had positive lesion uptake. Our patient underwent octreotide imaging when the diagnosis was unclear, which resulted in a high suspicion of PMT. Recently, a meta-analysis showed that 68Ga-DOTA-SST PET/CT is more effective than octreoscan-SPECT/CT for diagnosing PMTs.^[43]^ However, its high cost limits widespread application.^[44]^ Our patient had significant bone pain, multiple fractures, difficulty walking, and other symptoms of osteomalacia. Biochemical tests revealed a significant decrease in serum phosphate levels and an increase in the ALP levels; FGF23 levels were much greater than normal. 18F-OC PET/CT revealed a high possibility of PMT, and the patient was diagnosed with PMT. However, the most definitive diagnosis relies on pathological examination.

In 1987, Weidner et al classified PMTs into 4 pathological types: phosphaturic mesenchymal tumor, mixed connective tissue type (PMTMCT); phosphaturic mesenchymal tumor, osteoblastoma-like; phosphaturic mesenchymal tumor, non-ossifying fibroma-like; phosphaturic mesenchymal tumor, ossifying fibroma-like.^[37]^ Among these, PMTMCT is the most common subtype. In 2004, Folpe et al reviewed 32 new cases and concluded that there is essentially only one type of PMTMCT for PMTs.^[2]^ In 2019, Wu et al summarized the clinicopathological and immunohistochemical analyses of 22 patients with PMTs and proposed a new variant termed phosphaturic mesenchymal tumor, mixed epithelial, and connective tissue type (PMTMECT).^[45]^ However, to our knowledge, there is no definitive pathological diagnosis for PMTs to date.

Although PMTs are rare diseases, most of them are benign, and surgical resection of the tumor is the first choice of treatment for PMTs, which not only improves abnormalities in biochemical parameters but also resolves osteomalacia and promotes bone substance remineralization. The degree of surgical resection is correlated with tumor recurrence and symptom persistence. Complete tumor resection is the only treatment available to cure PMTs, and wide-margin tumor resection is necessary to reduce recurrence.^[41]^ Postoperative serum phosphate and FDF23 levels can quickly recover with remarkable clinical results. However, incomplete tumor resection can lead to local recurrence and recovery of related symptoms.^[46]^ Radiotherapy or radiofrequency ablation (RFA) can be used as an adjunctive treatment when the tumor cannot be completely resected for various reason.^[47,48]^ Medication therapy (oral supplementation with phosphates and 1,25(OH)2D3) is required for patients with undetected tumors or tumors that cannot be completely resected.^[49,50]^ For patients with disease progression or recurrence, new therapies, such as human monoclonal anti-FGF-23 antibodies, KRN23, somatostatin analogs and peptide receptor radionuclides, can be applied.^[51]^ Burosumab, a fully human monoclonal antibody against FGF23, can normalize phosphate metabolism by blocking excess FGF23. Recent studies have shown that Burosumab is superior to conventional medications in terms of efficacy, and can improve calcification. For patients whose tumors cannot be removed, are localized, or whose risk of surgery is too high, Burosumab may be an effective alternative to conventional treatment.^[52,53]^ Burosumab was approved by the FDA for the treatment of TIO in children and adults.^[54]^ In intracranial PMTs, tumors are adjacent to important structures, blood vessels, and nerves, and it is sometimes difficult to achieve complete resection. Therefore, adjunctive postoperative treatment is important. In our review, the recurrence rate was significantly lower (14.3%) following total resection compared to subtotal resection (50%). The 4 patients who relapsed after subtotal tumor resection received adjunctive therapies with drugs, stereotactic gamma knife radiosurgery and octreotide, PRRT, and local radiotherapy. Among these patients, all but one had a favorable prognosis. The patient who received postoperative local radiotherapy succumbed to multiple recurrences and disease progression. Therefore, when complete resection of intracranial PMTs is unattainable, neurosurgeons should proactively seek adjunctive therapies with drugs, octreotides, and radiotherapy to reduce the risk of tumor recurrence.

## 5. Limitation

Owing to the rarity of PMTs, most available studies are case reports, and large-scale clinical studies are lacking. Surgical resection is the preferred treatment option; however, the efficacy of various adjunctive therapeutic modalities has not been clearly recognized. Our review highlighted significant variability in the documentation of biochemical parameters, hindering effective comparative analysis of intracranial PMTs at different sites. Therefore, additional clinical studies are required for developing standardized treatment protocols. Detailed reporting of all laboratory and clinical indicators is critical to advance the understanding of PMTs.

## 6. Conclusion

PMTs are rare diseases that can occur in any part of the body but rarely affect the intracranial cavity. Intracranial PMTs should be strongly suspected in patients presenting with hypophosphatemia, osteomalacia, and intracranial occupancy. PET/CT imaging is an effective diagnostic tool for identifying potential PMTs. The current standard of care is complete tumor resection, which is he only definitive treatment for PMTs. If the patient’s general condition permits, total lesion excision should be performed. Medication and radiation therapy can be used as adjunct therapies. Therefore, early diagnosis and treatment should be considered to improve the prognosis of patients with PMTs.

## Author contributions

**Conceptualization:** Yujing Zhao, Zhe Wang.

**Data curation:** Shuyue Song, Yuyang Zhao, Yiquan Wang, Wenqiang Liu.

**Project administration:** Zhe Wang.

**Software:** Shuyue Song, Yuyang Zhao, Yiquan Wang, Wenqiang Liu.

**Writing – original draft:** Shuyue Song, Yuyang Zhao, Wenqiang Liu.

**Writing – review & editing:** Yiquan Wang, Yujing Zhao.
